# Spatial Heterogeneity in Light Supply Affects Intraspecific Competition of a Stoloniferous Clonal Plant

**DOI:** 10.1371/journal.pone.0039105

**Published:** 2012-06-13

**Authors:** Pu Wang, Jing-Pin Lei, Mai-He Li, Fei-Hai Yu

**Affiliations:** 1 College of Nature Conservation, Beijing Forestry University, Beijing, China; 2 Research Institute of Forestry, Chinese Academy of Forestry, Beijing, China; 3 Swiss Federal Institute for Forest, Snow and Landscape Research, Birmensdorf, Switzerland; Ohio State University, United States of America

## Abstract

Spatial heterogeneity in light supply is common in nature. Many studies have examined the effects of heterogeneous light supply on growth, morphology, physiology and biomass allocation of clonal plants, but few have tested those effects on intraspecific competition. In a greenhouse experiment, we grew one (no competition) or nine ramets (with intraspecific competition) of a stoloniferous clonal plant, *Duchesnea indica*, in three homogeneous light conditions (high, medium and low light intensity) and two heterogeneous ones differing in patch size (large and small patch treatments). The total light in the two heterogeneous treatments was the same as that in the homogeneous medium light treatment. Both decreasing light intensity and intraspecific competition significantly decreased the growth (biomass, number of ramets and total stolon length) of *D. indica*. As compared with the homogeneous medium light treatment, the large patch treatment significantly increased the growth of *D. indica* without intraspecific competition. However, the growth of *D. indica* with competition did not differ among the homogeneous medium light, the large and the small patch treatments. Consequently, light heterogeneity significantly increased intraspecific competition intensity, as measured by the decreased log response ratio. These results suggest that spatial heterogeneity in light supply can alter intraspecific interactions of clonal plants.

## Introduction

Spatial heterogeneity in light supply is common in natural habitats [1]–[3], and connected individuals (ramets) of clonal plants often grow across patches differing in light supply [4]–[6]. Although many studies have shown that spatial heterogeneity in light supply can affect the growth, morphology, physiology and/or biomass allocation of clonal plants [7]–[17], little is known about the effects of heterogeneous light supply on the interactions between clonal plants.

A few studies addressed the effects of spatial heterogeneity in soil nutrients on interactions between plants, and the results differed [18]–[22]. Soil nutrient heterogeneity increased intraspecific competition in *Briza media* [19] and interspecific competition between *Festuca rubra* and *Anthoxanthum odoratum* [20] and between *F. ovina* and *B. media* [19], but did not affect intraspecific competition in *F. ovina* [19] and interspecific competition between *Achillea millefolium* and six other species [21]. Spatial heterogeneity in soil nutrients was also found to change the relative abundance of species grown in mixtures [3], [22], [23]. So far, however, no study has tested the effects of spatial heterogeneity in light supply on intraspecific competition of clonal plants.

When a clone grows in environments with heterogeneous light supply consisting of low and high light patches, connected ramets growing in patches with different levels of light supply may exchange carbohydrates through clonal integration driven by source-sink relations [5], [7]–[11], [13], [17], [24], [25]. Consequently, performance of the ramets growing in low light patches may be greatly enhanced [4]–[10], [14], [18]. In some cases, such a support to the ramets in low light patches does not impose any costs on their connected ramets growing in high light patches, or the benefits of clonal integration to the ramets in low light patches are significantly larger than the costs to the ramets in high light patches [5], [11]. As a result, clonal integration greatly increases performance of the whole clone [5], [11]. Studies have shown that different clones of the same plant species may differ greatly in the ability of clonal integration under heterogeneous light supply [7]–[9], [13]. When a number of clones that differ in the ability of clonal integration grow in the same heterogeneous light environment, performance of clones may differ greatly due to the differences in the ability of clonal integration. In this case, light heterogeneity may change the intraspecific interactions of clonal plants.

Patch scale is an important element of spatial heterogeneity, and may have a substantial effect on performance of clonal plants [26]–[28]. For instance, *Glechoma hederacea* clones grown in heterogeneous conditions with the intermediate patch size (25 cm×25 cm) produced nearly four times as much biomass as those grown in heterogeneous environments with the smallest patch size (6.25 cm×6.25 cm) [28]. Therefore, clonal plants that respond to spatial heterogeneity in resource supply at one spatial scale may not do so at other scales [27]–[28]. This suggests that patch scale of light heterogeneity may also affect plant-plant interactions, i.e., light heterogeneity that affects intraspecific interactions at one scale may not at other scales. However, this hypothesis remains untested.

To address the effects of spatial heterogeneity in light supply on intraspecific competition, we conducted a greenhouse experiment with a stoloniferous herb *Duchesnea indica.* We grew one (no competition) or nine (with intraspecific competition) ramets of *D. indica* under three homogeneous light treatments (high, medium and low light intensity) and two heterogeneous light treatments differing in patch scale (large and small patch treatments). Specifically, we address the following questions: (1) Do light intensity in homogeneous conditions, light heterogeneity, and plant density (intraspecific competition) affect the growth of *D. indica*? We predicted that both decreasing light intensity and intraspecific interaction would decrease plant performance. We also predicted that light heterogeneity would increase plant performance when there was no competition because offspring ramets located in high light patches may support ramets located in low light patches at no or very low costs. (2) Does light intensity and heterogeneity affect intraspecific competition? We expected that both increasing light intensity and heterogeneity would increase intraspecific competition. (3) Does the scale of light heterogeneity matter? We predicted that effects of light heterogeneity on plant performance and intraspecific competition would depend on the patch scale, i.e., heterogeneity affect plant performance and intraspecific competition at one scale may not at another scale.

## Materials and Methods

### Plant species and experimental material

*Duchesnea indica* Focke is a perennial rosette herb belonging to the *Rosaceae* family, and distributed mainly in Asia [29]. This species occurs in many regions in China. It produces red fleshy fruits and compound leaves usually consisting of a slender petiole and three leaflets. This species produce long stolons with rooted ramets on its nodes [8], [30]. Interconnected ramets are often located in heterogeneous light environments [31].

In March 2011, more than 420 similar-sized ramets of *D. indica* were collected from a stock population in a greenhouse at Forestry Science Co, Ltd. of Beijing Forestry University. The exact genotypic information of these ramets was not known, but they were originally propagated from a number of seedlings established from seeds collected in the wild (Yu-Bao Sun, personal communications). Therefore, the ramets most likely belong to a number of different genotypes. For the experiments, 420 ramets were used and all stolons (if any) were removed. All the ramets were standardized by removing all the leaves except the youngest three or four and by cutting the roots to 5 cm long. Then, 20 ramets were randomly selected and dry mass was determined after drying at 70°C for 48 h to obtain the pre-planting biomass value (0.062±0.013 g, mean ± SE, n = 20).

### Experimental design

**Figure 1:**
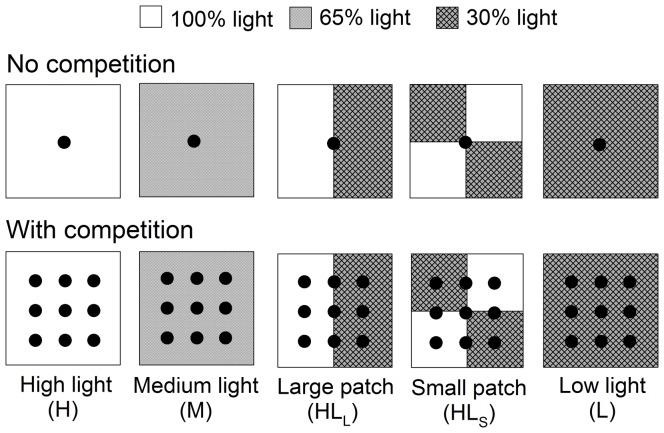
Experimental design. The experiment consisted of three homogeneous treatments (High light – the plants received full light in the greenhouse, coded as “H”; Medium light – the plants received 65% of full light, coded as “M”; Low light – the plants received 30% of full light, coded as “L”) and two heterogeneous treatments (Large patch, coded as “HL_L_” – the whole container was divided into two large patches; one patch received full light and the other 30% of full light; Small patch, coded as “HL_S_” – the whole container was divided into four small patches; two patches received full light and the other two 30% of full light), fully crossed with two treatments of competition (No competition – one plant per container; With competition – nine plants per container). The light received by the plants in the two patchy treatments was the same as that in the homogeneous medium light treatment.

The experiment was conducted in a heated glasshouse at the Institute of Botany, the Chinese Academy of Sciences in Beijing. There were ten treatments, i.e., two competition treatments (with or without intraspecific competition) fully crossed with three homogeneous light treatments (H, M and L for high, medium and low light intensity, respectively) and two heterogeneous light treatments differing in patch size (HL_L_ and HL_S_ for large and small patch treatments, respectively). In the competition-free treatments, one ramet of *D. indica* was planted in the center of each plastic container (34 cm long×34 cm wide), whereas in the competition treatments, nine ramets were planted within each container (Fig. 1). Ramets in the containers received full light in the glasshouse in the homogeneous high light treatment (H), and 65% and 30% of full light in the homogeneous medium (M) and low (L) light treatment, respectively. The treatments of M and L were applied by covering the containers with two types of black, neutral shading net without changing the red light to far red light ratio. In the large patch treatment (HL_L_) each container was divided into two large patches and in the small patch treatment (HL_S_) each divided into four small patches (Fig. 1). In HL_L_, half of the containers (34 cm×17 cm in size) received full light, whereas the other half was covered using the shading net applied in L that allows 30% of the full light to pass through. In HL_S_, the shading net covering the container was divided into four 17 cm×17 cm patches, and two of them were removed (and the edges were fixed with wires) so that 100% of full light could pass through and the other two were not so that 30% of full light could pass through. Therefore, the total amount of light received by plants in the two patchy treatments was the same as that in M.

The growth substrate in each container was a 25-cm-deep, 1∶1 (v∶v) mixture of washed river sand and commercial peat (Screening: 0–10 mm; NPK fertilizer, Mg and micro nutrients are added). There were eight replicates in each treatment.

The experiment lasted from 4 March to 31 May 2011. During the experiment the mean temperature in the greenhouse was set to 25°C and the relative humidity to 65%. The light intensity in the greenhouse was about 60% of the outside, and no additional artificial light was provided. Tap water was supplied regularly to ensure there was sufficient water for plants to grow. The containers were randomly placed within a small area of about 25 m^2^ and all containers were rotated horizontally for 180° to avoid potential effects of positions.

### Harvest and measurements

During the experiment each initial (parent) ramet produced a number of offspring ramets that were confined within the containers and allowed to root. At harvest, parent ramets and offspring ramets were harvested separately. For the two heterogeneous treatments, we harvested offspring ramets located in the high light patches and those located in the low light patches separately. We counted number of all ramets (parent plus offspring ramets) and measured stolon length. Then, parent ramets and offspring ramets were oven-dried at 70°C for 48 h, and weighed.

### Data analysis

Before analysis, values of all variables in the competition treatments (i.e., with nine ramets per container) were divided by nine so that the values were scaled to the level of per initial plant. To measure the intensity of intraspecific interactions, we calculated the log response ratio [32] as: LnRR = ln(B_+_/B_0_), where LnRR is the log response ratio, B_+_ is biomass per initial plant with competition and B_0_ is the mean biomass per initial plant without competition across the eight replicates. Values of LnRR are symmetrical around zero, with negative values indicating competition and positive values indicating facilitation [33].

Two-way ANOVAs were used to test the effects of competition (with and without), light conditions (H, M, L, HL_S_ and HL_L_) and their interactions on biomass, number of ramets and stolon length per initial plant per container. When significant effects were found, Duncan multiple comparison tests were conducted to examine for differences between the ten treatments [34]. We used one-way ANOVA followed by Duncan tests to compare the means of LnRR among the five light treatments.

To test the effects of competition and light heterogeneity on the growth measures of the plants in the high light patches, we used two-way ANOVAs. In these analyses, the growth measures of the plants in the high light patches in the heterogeneous treatments (HL_L_ and HL_S_) and 50% of the value of each growth measure of the plants in the homogeneous high light treatments (H) were used. We used 50% of the values in H because the area with high light in H was two times of that in the HL_L_ or HL_S_. Similarly, we tested the effects of competition and light heterogeneity on the growth measures of the plants in the low light patches. In these analyses, the growth measures of the plants in the low light patches in HL_L_ and HL_S_, and 50% of the value of each growth measure of the plants in L were used. If significant effects were detected, then Duncan multiple comparison tests were used to compare the means between the treatments.

All analyses were conducted with SPSS 17.0 software (SPSS, Chicago, IL, USA). Prior to ANOVAs, all data were checked for normality and homoscedasticity. The differences were considered to be significant if *P*<0.05.

## Results

### Effects of competition and light intensity at whole plant (container) level

**Figure 2:**
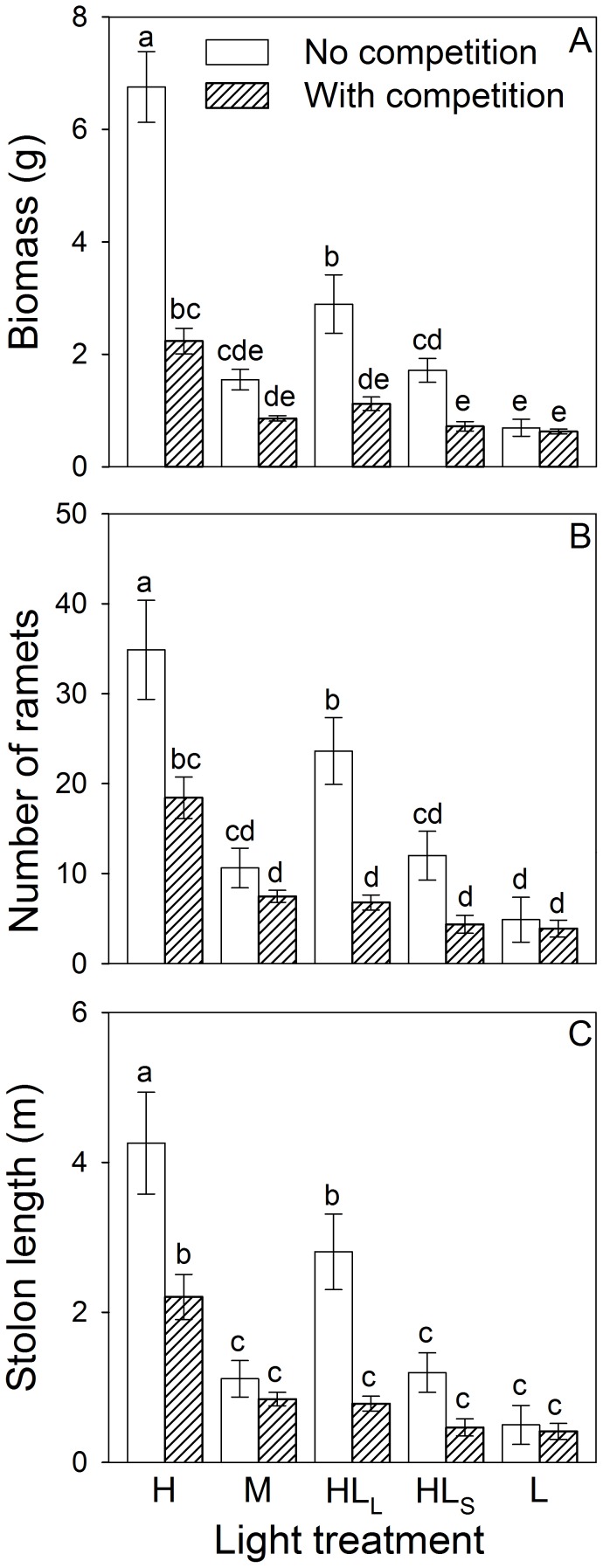
Effects of competition and light treatment on the whole clone of *Duchesnea indica*. Mean values (+SE) of biomass (A), number of ramets (B) and total stolon length (C) are given. Bars sharing the same letters are not different at *P* = 0.05. Treatment codes are in Figure 1.

**Figure 3:**
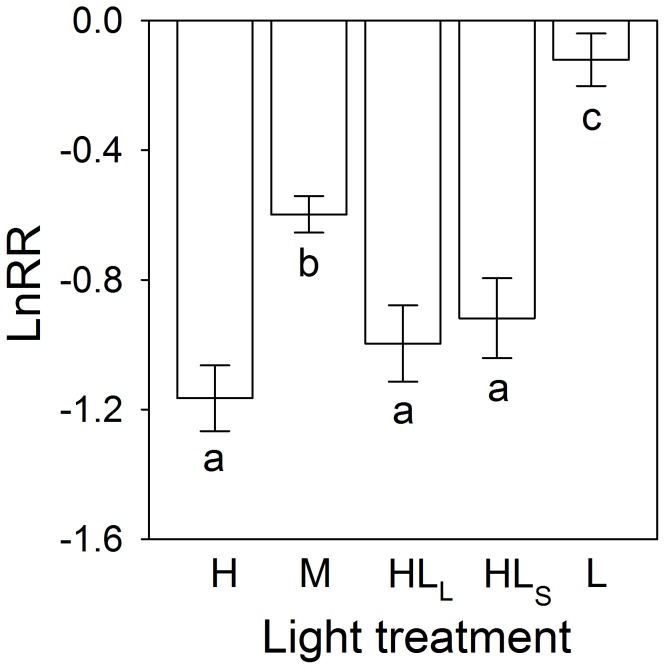
Effects of light treatment on competition intensity of *Duchesnea indica*. The competition intensity was measured by the log response ratio (LnRR) of biomass of the whole clone. Bars are mean values (+SE). Bars sharing the same letters are not different at *P* = 0.05. Treatment codes are in Figure 1.

Under homogeneous treatments, decreasing light intensity significantly decreased biomass, number of ramets and total stolon length of *D. indica* (Fig. 2; Table 1). The presence of neighbors significantly decreased these three growth measures in the high light treatments, but not in the medium or low light conditions (Fig. 2). Decreasing light intensity significantly decreased competition intensity as measured by the increased log response ratio of biomass (Fig. 3).

**Table 1:** ANOVAs for effects of competition and light treatments (intensity and heterogeneity) on the growth measures of *Duchesnea indica* at whole plant level.

Effect		Biomass		No. of ramets		Stolon length	
	DF	*F*	*P*	*F*	*P*	*F*	*P*
Competition (C)	1,70	76.9	<0.001	28.4	<0.001	25.6	<0.001
Light (L)	4,70	53.5	<0.001	21.4	<0.001	23.4	<0.001
C×L	4,70	18.2	<0.001	3.8	0.007	4.3	0.004

**Table 2:** ANOVAs for effects of competition and light heterogeneity on the growth measures of *Duchesnea indica* in the high (A) and low light patches (B).

Effect		Biomass		No. of ramets		Stolon length	
	DF	*F*	*P*	*F*	*P*	*F*	*P*
(A) High light							
Competition (C)	1,42	50.7	<0.001	20.0	<0.001	18.2	<0.001
Heterogeneity (H)	2,42	50.0	<0.001	14.5	<0.001	16.3	<0.001
C×H	2,42	15.6	<0.001	0.5	0.593	0.9	0.426
(B) Low light							
Competition (C)	1,42	15.8	<0.001	14.1	0.001	13.9	0.001
Heterogeneity (H)	2,42	12.4	<0.001	6.0	0.005	12.1	<0.001
C×H	2,42	9.1	0.001	3.9	0.029	7.5	0.002

### Effects of competition and light heterogeneity at whole plant (container) level

Without competition all three growth measures of *D. indica* in the large patch treatment (HL_L_) were significantly greater than those in the homogeneous medium light treatment (M). With competition, however, these growth measures did not differ significantly between M and HL_L_ (Fig. 2; Table 1). No matter whether there was competition or not, none of the three growth measures differed significantly between M and the small patch treatment (HL_S_; Fig. 2). Log response ratio was negative and significantly larger in M than in the two heterogeneous treatments (HL_L_ and HL_S_; Fig. 3), and it did not differ significantly between HL_L_ and HL_S_ (Fig. 3).

### Effects of competition and light heterogeneity at patch level

**Figure 4:**
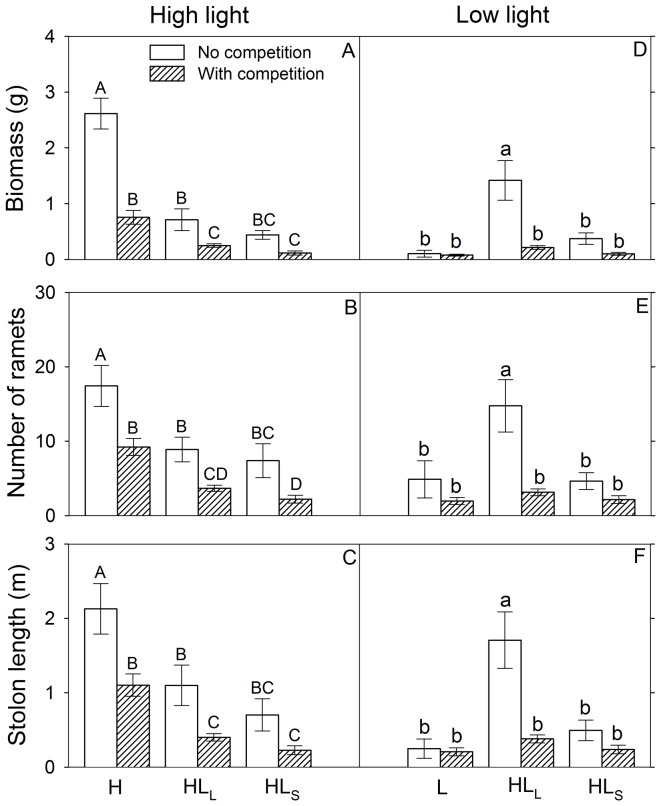
Effects of competition and light heterogeneity on growth measures of *Duchesnea indica*. Bars in the left panels (A–C) are mean values (+SE) of three growth measures in the high light patches and bars in the right panels (D–F) are those in the low light patches. Bars sharing the same letters are not different at *P* = 0.05. Bars are mean values (+SE). Treatment codes are in Figure 1.

No matter whether there was competition or not, all three growth measures in the high light patches were significantly smaller in HL_L_ or HL_S_ than in the comparable area (half a container) of H (Fig. 4A, B and C, Table 2). Without competition all three growth measures in the low light patches were markedly larger in HL_L_ than in L or HL_S_, whereas with competition none of these growth measures differed significantly among L, HL_L_ and HL_S_ (Fig. 4D, E and F). Competition greatly decreased all growth measures in HL_L_, but did not significantly affect the growth in L or HL_S_ (Fig. 4D, E and F).

## Discussion

Decreasing light intensity significantly decreased intraspecific competition intensity of *D. indica*. Many studies have shown that competition becomes more intense when resource supply is higher [19], [35], [36]. The likely reason is that under high resource conditions (e.g., high light conditions in the present study) plants will grow vigorously so that they strongly compete for light, nutrients and/or water, but under very low resource conditions, plants grow so weakly that they do not need to compete for such resources [37]. This explanation is supported by the fact that under high light conditions the presence of neighbors significantly decreased the growth of *D. indica* but under low light conditions it did not.

Studies generally show positive effects of spatial heterogeneity in resource supply on the growth of single clones [8], [11], [38]–[43]. For instance, clones of *G. hederacea* grown in heterogeneous conditions in soil nutrients produced over 1.5 times more biomass than those in homogeneous conditions [39], and clones of *Potentilla reptans* grown in reciprocally or coincidentally patchy conditions both accumulated 70% more biomass than those grown in homogeneous conditions [11]. The underlying mechanism is very likely that in heterogeneous conditions the concentration of ramets, roots or leaves in resource-rich patches allows clones to highly efficiently uptake resources and such resources are re-distributed within the clones through physiological integration to increase the growth of the whole clones [11], [39].

We also found that *D. indica* without intraspecific competition grew more when light availability was spatially heterogeneous than when it was homogeneous (Fig. 2). During the experiment, the parent ramets of *D. indica* planted at the borders between high and low light patches produced many offspring ramets that were located either in high light patches or in low light ones (P Wang personal observation). We found that the growth of *D. indica* in the high or low light patches of the heterogeneous treatments differed greatly from that in the corresponding areas of the homogeneous treatments (Fig. 4). These results suggest that clonal integration (most likely for carbohydrates) was likely to occur among interconnected parent ramets in the patch borders and offspring ramets located in the high or low light patches in the heterogeneous light treatments [5], [44]. Such clonal integration may have markedly increased the growth of the plants in the low light patches, and further led to the increased growth of the whole plant in the large patch treatment without competition (Fig. 2).

However, when there was strong intraspecific competition, the growth of *D. indica* could not benefit from spatial heterogeneity in light supply, which has not been reported before. The reason might be that, when growing in heterogeneous conditions, all clones will concentrate their leaves or offspring ramets in the high light patches [4], [11], [39], [45]. This may result in great costs for intraspecific competition [37], and thus plants of *D. indica* with intraspecific neighbors could not benefit from light heterogeneity.

Light heterogeneity significantly increased the intraspecific competition intensity of *D. indica.* One explanation is that the ability of physiological integration and thus the ability to selectively place offspring ramets differ greatly among the genotypes of *D. indica*. When plants with different ability of physiological integration and/or morphological plasticity are grown in the same heterogeneous environment, the beneficial effects of heterogeneity on the growth will differ among plants [7], [16]. As a result, heterogeneity will change the intraspecific competition intensity. Another explanation is that when growing in heterogeneous conditions all plants will concentrate their leaves in the high light patches, which greatly increases the intensity of intraspecific competition [19], [37], [46]–[48].

Our results suggest for the first time that spatial heterogeneity in light supply can change intraspecific interactions of clonal plants. Therefore, spatial heterogeneity in light supply may be of great importance in regulating population structure and dynamics of clonal plants [11], [19], [46], [47], [49].
